# Associations of Pretransplant Patient-Reported Outcomes Measurement Information System Physical Function Score With Kidney Transplant Outcomes

**DOI:** 10.3389/ti.2025.13884

**Published:** 2025-01-29

**Authors:** Junji Yamauchi, Amy M. Cizik, Katalin Fornadi, Dominik Thomas, Divya Raghavan, Duha Jweehan, Suayp Oygen, Silviana Marineci, Michelle Buff, Motaz Selim, Michael Zimmerman, Istvan Mucsi, Miklos Z. Molnar

**Affiliations:** ^1^ Department of Internal Medicine, Division of Nephrology and Hypertension, Spencer Fox Eccles School of Medicine at the University of Utah, Salt Lake City, UT, United States; ^2^ Department of Orthopedics, Spencer Fox Eccles School of Medicine at the University of Utah, Salt Lake City, UT, United States; ^3^ Department of Surgery, Division of Transplantation and Advanced Hepatobiliary Surgery, Spencer Fox Eccles School of Medicine at the University of Utah, Salt Lake City, UT, United States; ^4^ Temerty Faculty of Medicine, University of Toronto, Toronto, ON, Canada; ^5^ Ajmera Transplant Centre, University Health Network, Toronto, ON, Canada

**Keywords:** kidney transplantation, transplant outcomes, PROMIS^®^, Patient-Reported Outcomes Measurement Information System^®^, physical function

## Abstract

Simple and validated physical function measures are needed for kidney transplant candidates because pretransplant low physical function is a common and potentially modifiable risk factor. This single-center retrospective study investigated the associations between pretransplant physical function assessed by the Patient-Reported Outcomes Measurement Information System^®^ Physical Function (PROMIS-PF) computer adaptive testing and early posttransplant outcomes. We analyzed 154 adult kidney-alone transplant recipients. The median pretransplant PROMIS-PF score was 43 (interquartile range, 39–47). Patient characteristics were not significantly different across the score category (normal, score ≥45; mild, score of 40–45; and moderate/severe, score <40). The PROMIS-PF score was not associated with length of transplant hospital stay, delayed graft function, 6-month and 12-month graft function, or 12-month patient and graft survival. However, a lower PROMIS-PF score was significantly associated with a higher risk of emergency room visits [adjusted odds ratios compared to normal: mild, 1.68 (95% confidence interval, 0.76–3.83); moderate/severe, 3.23 (1.34–7.79)] and rehospitalization [adjusted odds ratios: mild, 2.61 (1.16–5.90); moderate/severe, 2.53 (1.07–6.00)] within 1 month posttransplant. Results suggest that PROMIS-PF is a practical tool for assessing physical function in kidney transplant candidates. Larger studies are needed to confirm the utility of PROMIS-PF to identify transplant candidates who would benefit from pretransplant prehabilitation.

## Graphical Abstract

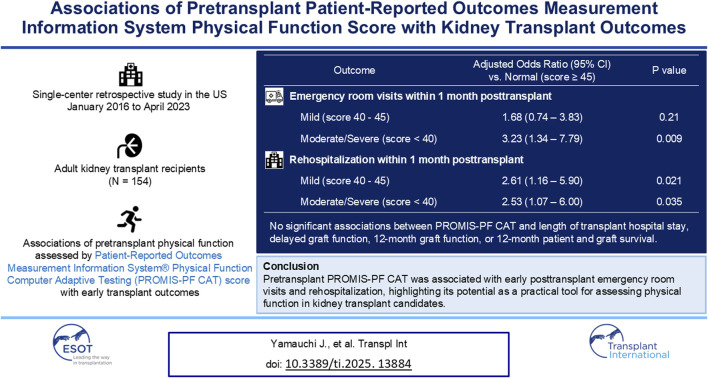

## Introduction

Low physical function is common among individuals with kidney failure and is associated with poor prognosis after kidney transplantation [1–5]. Therefore, physical function assessment may help identify kidney transplant recipients who might be candidates for pretransplant rehabilitation (prehabilitation). However, performance-based physical function assessments, such as the short physical performance battery or the 6-Minute Walk Test, require training to administer and can be time-consuming, more efficient, validated, assessment tools are needed for daily clinical use [6]. Patient-reported outcome measures (PROMs), such as the 36-item Short Form Health Survey (SF-36), are also utilized to evaluate physical function [7]. Although they are easier to administer than physical performance tests, the burden of completing extensive questionnaires remains a significant barrier to widespread use. Furthermore, the reliability of these tools is limited in patients with markedly below-average physical functioning [8].

The Patient-Reported Outcomes Measurement Information System (PROMIS^®^) was developed with the support of the National Institutes of Health to establish standardized, generic patient-reported outcome measures [9]. PROMIS offers fixed-length testing and computer adaptive testing (CAT). In fixed-length short form testing, using 4-item or 8-item short forms, a predetermined set of questions is administered irrespective of the respondent’s functional status. Conversely, PROMIS CAT utilizes item banks and administers questions that are optimized by item response theory (IRT) and selected based on previous answers using score estimation algorithms [10]. With CATs, all participants begin with the first item, targeting the midpoint of the T-score (functional) range. Subsequent items are selected by an algorithm based on responses to previous items until a stopping rule (reliability >90% or completing 12 items) is satisfied. PROMIS CAT requires fewer questions compared to other PROMs not developed using IRT, thereby substantially reducing the question burden. The CAT system can yield highly precise results with an average of only 4–6 questions. PROMIS CAT and short forms produce comparable scores [11].

PROMIS Physical Function (PROMIS-PF) measures the domain of physical function and has been validated in several disease conditions including chronic kidney disease [12–15]. A recent study found that a lower pretransplant PROMIS-PF 4-item short form score was significantly associated with a higher risk of rehospitalization within 1 month after kidney transplantation [16]. However, the utility of pretransplant PROMIS-PF CAT assessments or their associations with posttransplant outcomes have not been evaluated in kidney transplant recipients. We therefore investigated the associations of the pretransplant PROMIS-PF CAT scores with transplant outcomes within 12 months posttransplant. Our hypothesis was that a lower pretransplant PROMIS-PF CAT score is associated with worse early posttransplant outcomes, such as higher hospitalization rates and longer transplant hospital stays.

## Materials and Methods

### Data Source and Study Population

This retrospective study included adult kidney-alone transplant recipients who underwent transplantation at the University of Utah hospital from January 2016 to April 2023 and received a PROMIS-PF CAT within 12 months pretransplant. Recipients less than 18 years of age or those who underwent multi-organ transplantation were excluded. Patient data were extracted from our enterprise data warehouse. This study was approved by the University of Utah Institutional Review Board (IRB_00162331), which also granted an exemption from informed consent.

### Measurement and Interpretation of the PROMIS-PF Score

PROMIS-PF item banks version 1.2 or version 2.0 were administered as CAT at outpatient clinics for non-research, clinical purposes using our proprietary university-developed system, My Evaluation (mEVAL), which was introduced at University of Utah Health in 2015 to facilitate standardized PROM assessments across various care settings [17]. The PROMIS-PF item banks consist of 165 items across four subdomains: instrumental activities of daily living, mobility or lower extremity function, back and neck (central) function, and upper extremity function [18]. Responses to the items range from 1 (“cannot do”) to 5 (“not at all” or “without any difficulty”). PROMIS-PF was scored using the T-score metric. The PROMIS-PF score ranges from 20 points to 80 points, with the US general population mean ± standard deviation of 50 ± 10. A higher score indicates better physical function. The PROMIS scoring guidelines classify PROMIS-PF scores into no significant physical function impairment (normal, score ≥45), mild (40 to <45), moderate (30 to <40), and severe (<30) [19]. In the current study, PROMIS-PF scores were categorized into normal, mild, and moderate/severe because only six patients fell into the severe category. For patients with multiple measurements within 12 months preceding the index kidney transplantation, the PROMIS-PF score closest to the transplant date was used for analysis. We did not perform the psychometric property testing because it has been already established in the chronic kidney disease population [12].

### Outcomes

The outcomes of interest were associations between the pretransplant PROMIS-PF score and early post-transplant outcomes, including length of transplant hospital stay (LOS), delayed graft function defined as any dialysis in the first week post-transplant, emergency room visits and rehospitalization for any reason within 1 month posttransplant, 6-month and 12-month estimated glomerular filtration rate (eGFR) calculated via the Chronic Kidney Disease Epidemiology Collaboration equation 2021 [20], and 12-month patient and graft survival. We collected data on emergency room visits and rehospitalizations to our hospital because our clinical protocol required all recipients to remain near our hospital and contact us directly during the first month of post-transplant. All rehospitalizations were included regardless of the length of hospital stay.

### Covariates for Multivariable Regression Analysis

For multivariable linear and logistic regression analyses, we selected covariates based on published literature and theoretical considerations [16, 21, 22]. We adjusted for donor factors (age, donor type, donation after brain death/circulatory death, and cold ischemia time) and recipient variables (age, sex, race, Charleson Comorbidity Index [23, 24], prior organ transplant, preemptive transplant, calculated panel reactive antibody, and lymphocyte-depleting antibody induction). In the logistic regression for emergency room visits and rehospitalization, due to the limited number of events, we first calculated propensity scores for each outcome using all the covariates and then calculated odds ratios, adjusting only for the propensity score.

### Statistical Analysis

We used mean ± standard deviation or median and interquartile range (IQR) for summarizing continuous variables and number (%) for categorical variables. Patient characteristics at transplantation and observed outcomes were delineated in accordance with the PROMIS-PF score category (normal, mild, and moderate/severe). We used the Jonckheere–Terpstra trend test to analyze the trends of baseline characteristics and outcomes across the PROMIS-PF score category. We used linear regression to analyze the associations of the PROMIS-PF score with LOS and 6-month and 12-month eGFR. Logistic regression was used to analyze the associations of the PROMIS-PF score with the presence/absence of emergency room visits and rehospitalization within 1 month posttransplant. We used the two-sided p-value of <0.05 to adjudicate statistical significance. STATA Version 18 was used for all statistical analyses (STATA Corporation, College Station, TX).

## Results

### Patient Characteristics

**FIGURE 1:**
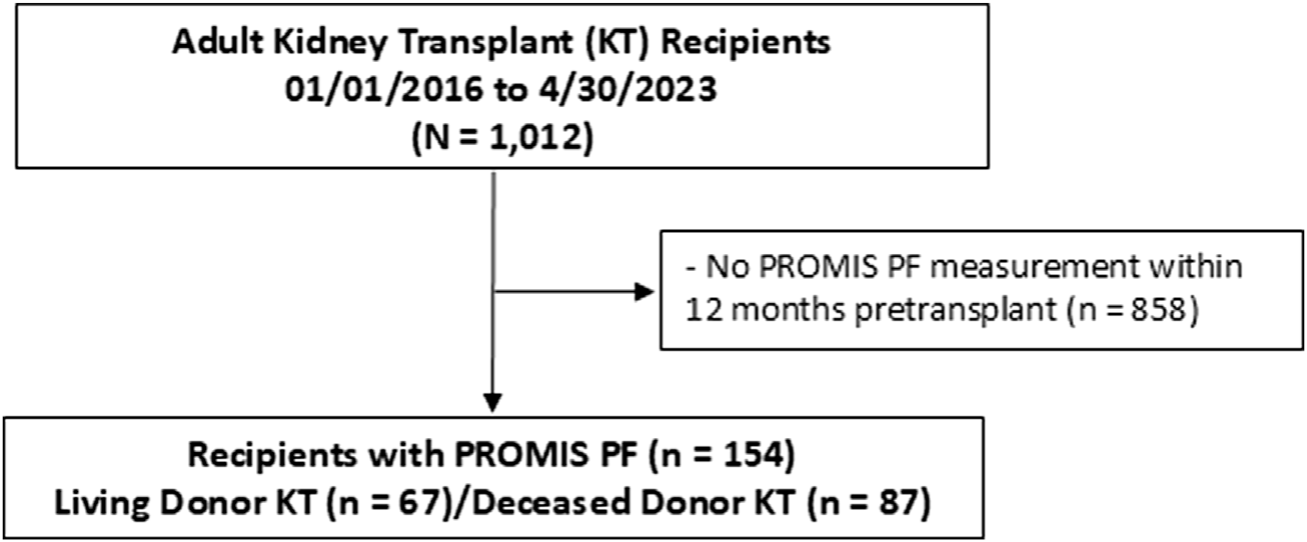
Study flowchart. PROMIS PF, Patient-Reported Outcomes Measurement Information System Physical Function.

Among 1,012 kidney transplant recipients, a total of 154 kidney recipients had the PROMIS-PF score evaluated within 12 months pretransplant (Figure 1). The median number of PROMIS-PF tests was 1 (IQR, 1–2), with 113 recipients (73%) undergoing one assessment within 12 months pretransplant. PROMIS-PF was assessed at a median of 5 (IQR, 3–8) months prior to transplantation. The median number of questions answered was 4 (IQR, 4–4), with 139 recipients (90%) required to answer four questions. The maximum number of questions answered was 11. Table 1 shows patient characteristics at kidney transplantation according to the pretransplant PROMIS-PF score category [normal (n = 61, 40%), mild (n = 52, 34%), and moderate/severe (n = 41, 27%)]. Median pretransplant PROMIS-PF score was 43 (IQR, 39–47). Recipients had a mean age of 52 ± 14 years; 41% were female; and the majority were white (68%). History of diabetes was reported in 38% and 32% had kidney failure from diabetes. Median Charleson Comorbidity Index was 5 (IQR, 3–6). Additionally, 25% underwent preemptive transplant. Lymphocyte-depleting antibody induction (anti-thymocyte globulin or alemtuzumab) was administered to 62% of recipients. The majority received tacrolimus (91%), mycophenolate (100%), and steroids (100%) as maintenance immunosuppression at transplant hospitalization discharge. Donors were living in 44%, with a mean age of 39 ± 15 years, and 50% were female. Overall, no significant trends were found in recipient and donor characteristics across the PROMIS-PF category.

**TABLE 1:** Patient characteristics.

Characteristic		PROMIS PF category			p for trend
	Total	Normal (≥45)	Mild (40–<45)	Moderate/Severe (<40)	
	N = 154	N = 61	N = 52	N = 41	
Recipient					
Pretransplant Physical Function score	43 (39–47)	48 (46–52)	41 (40–43)	36 (34–38)	<0.001
Number of PROMIS-PF questions answered	4 (4–4)	4 (4–4)	4 (4–4)	4 (4–4)	0.13
Months from pretransplant assessment to transplant	5 (8–3)	6 (8–4)	5 (8–2)	4 (7–3)	0.25
Age (years)	52 (14)	53 (15)	52 (15)	51 (14)	0.44
Sex					0.40
Female	63 (41%)	23 (38%)	21 (40%)	19 (46%)	
Male	91 (59%)	38 (62%)	31 (60%)	22 (54%)	
Body mass index (kg/m^2^)	28.2 (5.2)	27.3 (4.8)	28.8 (5.1)	28.7 (5.6)	0.15
Race					0.26^a^
White	105 (68%)	42 (69%)	35 (67%)	28 (68%)	
Black	5 (3%)	1 (2%)	0 (0%)	4 (10%)	
Hispanic	21 (14%)	7 (11%)	9 (17%)	5 (12%)	
Asian	5 (3%)	1 (2%)	3 (6%)	1 (2%)	
American Indian/Alaska Native	4 (3%)	1 (2%)	2 (4%)	1 (2%)	
Native Hawaiian/other Pacific Islander	13 (8%)	8 (13%)	3 (6%)	2 (5%)	
Multiracial	1 (1%)	1 (2%)	0 (0%)	0 (0%)	
History of diabetes	59 (38%)	23 (38%)	21 (40%)	15 (37%)	0.96
Prior organ transplantation	15 (10%)	9 (15%)	4 (8%)	2 (5%)	0.085
Dialysis duration					0.73
Preemptive	38 (25%)	15 (25%)	14 (27%)	9 (22%)	
≤1 year	25 (16%)	9 (15%)	10 (19%)	6 (15%)	
1–3 years	32 (21%)	13 (21%)	10 (19%)	9 (22%)	
3–5 years	27 (18%)	13 (21%)	7 (13%)	7 (17%)	
>5 years	32 (21%)	11 (18%)	11 (21%)	10 (24%)	
Cause of kidney failure					0.62^a^
Diabetes	50 (32%)	19 (31%)	19 (37%)	12 (29%)	
Hypertension	19 (12%)	6 (10%)	5 (10%)	8 (20%)	
Glomerulonephritis	22 (14%)	9 (15%)	5 (10%)	8 (20%)	
Cystic disease	27 (18%)	10 (16%)	11 (21%)	6 (15%)	
Others	36 (23%)	17 (28%)	12 (23%)	7 (17%)	
Hepatitis B virus core antibody	12 (9%)	9 (17%)	0 (0%)	3 (8%)	0.060
Hepatitis C virus antibody	5 (3%)	2 (3%)	1 (2%)	2 (5%)	0.74
Human immunodeficiency virus antibody	2 (1%)	0 (0%)	2 (4%)	0 (0%)	0.14
Charlson Comorbidity Index at transplant	5 (3–6)	5 (3–6)	5 (4–7)	5 (3–7)	0.55
Charlson Comorbidity Index category (tertile)					0.44
2–4	69 (45%)	30 (49%)	21 (40%)	18 (44%)	
5–6	47 (31%)	18 (30%)	17 (33%)	12 (29%)	
7–16	38 (25%)	13 (21%)	14 (27%)	11 (27%)	
Calculated panel reactive antibody (%)	0 (0–0)	0 (0–16)	0 (0–0)	0 (0–0)	0.35
Human leucocyte antigen mismatch					0.74
0	8 (5%)	5 (8%)	1 (2%)	2 (5%)	
1	7 (5%)	4 (7%)	1 (2%)	2 (5%)	
2	16 (10%)	8 (13%)	4 (8%)	4 (10%)	
3	23 (15%)	8 (13%)	7 (13%)	8 (20%)	
4	31 (20%)	10 (16%)	15 (29%)	6 (15%)	
5	49 (32%)	15 (25%)	19 (37%)	15 (37%)	
6	20 (13%)	11 (18%)	5 (10%)	4 (10%)	
Induction immunosuppression					
Lymphocyte depleting induction	95 (62%)	40 (66%)	29 (56%)	26 (63%)	0.69
Anti-thymocyte globulin	56 (36%)	20 (33%)	21 (40%)	15 (37%)	0.61
Alemtuzumab	41 (27%)	21 (34%)	8 (15%)	12 (29%)	0.34
Basiliximab	10 (6%)	3 (5%)	4 (8%)	3 (7%)	0.58
Maintenaice immunosuppression at discharge					
Tacrolimus	140 (91%)	57 (93%)	46 (88%)	37 (90%)	0.50
Cyclosporine	2 (1%)	1 (2%)	0 (0%)	1 (2%)	0.86
Everolimus	1 (1%)	0 (0%)	1 (2%)	0 (0%)	0.81
Belatacept	19 (12%)	6 (10%)	8 (15%)	5 (12%)	0.62
Mycophenolate	154 (100%)	61 (100%)	52 (100%)	41 (100%)	—
Steroids	154 (100%)	61 (100%)	52 (100%)	41 (100%)	—
Donor					
Donor type					0.80
Living donor	67 (44%)	27 (44%)	23 (44%)	17 (41%)	
Deceased donor	87 (56%)	34 (56%)	29 (56%)	24 (59%)	
Age (years)	39 (15)	39 (15)	38 (15)	41 (15)	0.62
Sex					0.90
Female	77 (50%)	29 (48%)	30 (58%)	18 (44%)	
Male	77 (50%)	32 (52%)	22 (42%)	23 (56%)	
Terminal serum creatinine (mg/dL)	0.96 (0.48)	0.90 (0.29)	0.99 (0.66)	1.00 (0.46)	0.44
Kidney Donor Profile Index^b^	39 (23)	39 (24)	35 (22)	46 (22)	0.34
Donation after circulatory death^b^	30 (34%)	11 (32%)	9 (31%)	10 (42%)	0.51
Donor kidney on-pump	86 (56%)	35 (57%)	28 (54%)	23 (56%)	0.85
Cold ischemia time (hours)	9 (8)	9 (8)	10 (9)	8 (8)	0.83

Recipients were stratified into three groups: no significant physical function impairment (PROMIS-PF score ≥45), mild (40–<45), and moderate/severe (<40). Values are expressed as mean (standard deviation), median (interquartile range), or number (%). The Jonckheere–Terpstra trend test was used to calculate p-values for trend.

^a^ P-values for race and cause of kidney failure were calculated via Chi-square tests.

^b^ Only for deceased donors.

PROMIS PF, Patient-Reported Outcomes Measurement Information System Physical Function.

### Early Posttransplant Outcomes

Table 2 summarizes the observed outcomes. The median LOS was 3 (IQR, 3–4) days. Delayed graft function was reported in 6 recipients (4%). The mean 6-month and 12-month eGFR were 63 ± 21 and 63 ± 20 mL/min/1.73 m^2^. Patient death and death-censored graft failure were reported in 10 (6%) and 2 (1%) recipients at 12 months. Biopsy-proven rejection and *de novo* donor-specific antibody class I and class II were observed in 15 (10%), 11 (7%), and 18 (12%) recipients at 12 months, respectively. There were no significant trends in these outcomes across the PROMIS-PF category. Emergency room visits and rehospitalization within 1 month posttransplant were observed in 58 (38%) and 59 (38%) recipients, respectively; and proportions of these outcomes were significantly higher in the mild and moderate/severe groups than the normal group (Jonckheere–Terpstra test for trend, p = 0.018 and 0.024, respectively). Reasons for emergency room visits and rehospitalizations are summarized in Supplementary Table S1. Infection was the most common reason for emergency room visits (n = 14, 9%), including urinary tract infections (n = 9, 6%) and other infections (n = 5, 3%). Surgical complications were the second most frequent cause (n = 13, 8%), primarily related to surgical wounds (n = 11, 7%) and other surgical complications (n = 2, 1%). Similarly, rehospitalization was most often due to infection (n = 16, 10%), with urinary tract infections (n = 8, 5%) being the primary contributor. Surgical complications were also the second leading cause of rehospitalization (n = 10, 6%), due to surgical wound problems (n = 6, 4%) and other complications (n = 4, 3%).

**TABLE 2:** Transplant outcomes according to the PROMIS-PF score category.

Outcome		PROMIS PF category			p for trend
	Total	Normal (≥45)	Mild (40–<45)	Moderate/Severe (<40)	
	N = 154	N = 61	N = 52	N = 41	
Length of hospital stay (days)	3 (3–4)	3 (3–4)	3 (3–4)	3 (3–4)	0.68
Length of hospital stay ≥7 days	13 (8%)	3 (5%)	7 (13%)	3 (7%)	0.49
Delayed graft function	6 (4%)	2 (3%)	3 (6%)	1 (2%)	0.95
Any emergency room visit within 1 month	58 (38%)	17 (28%)	20 (38%)	21 (51%)	**0.018**
Any rehospitalization within 1 month	59 (38%)	16 (26%)	24 (46%)	19 (46%)	**0.024**
6-month eGFR (mL/min/1.73 m^2^)	63 (21)	64 (18)	61 (22)	64 (24)	0.79
12-month eGFR (mL/min/1.73 m^2^)	63 (20)	62 (20)	62 (21)	66 (21)	0.63
6-month death-censored graft failure	1 (1%)	0 (0%)	1 (2%)	0 (0%)	0.81
12-month death-censored graft failure	2 (1%)	0 (0%)	2 (4%)	0 (0%)	0.73
6-month mortality	2 (1%)	1 (2%)	0 (0%)	1 (2%)	0.87
12-month mortality	10 (6%)	3 (5%)	5 (10%)	2 (5%)	0.85
Rejection within 12 months	15 (10%)	5 (8%)	7 (13%)	3 (7%)	0.96
*de novo* DSA class I within 12 months	11 (7%)	4 (7%)	4 (8%)	3 (7%)	0.86
*de novo* DSA class II within 12 months	18 (12%)	4 (7%)	9 (17%)	5 (12%)	0.26

Recipients were stratified into three groups: no significant physical function impairment (PROMIS-PF score ≥45), mild (40-<45), and moderate/severe (<40). Values are expressed as mean (standard deviation), median (interquartile range), or number (%). The Jonckheere–Terpstra trend test was used to calculate p-values for trend. Bold values denote statistically significant differences with p-values <0.05.

DSA, donor-specific antibody; PROMIS PF, Patient-Reported Outcomes Measurement Information System Physical Function.

### Associations of Pretransplant PROMIS-PF Score and Transplant Outcomes

In the multivariate regression analysis, the pretransplant PROMIS-PF score was not significantly associated with LOS or 6-month/12-month eGFR (Table 3; coefficients for covariates are available in Supplementary Tables S2–S4). However, significant associations were found between the pretransplant PROMIS-PF score and emergency room visits and rehospitalization within 1 month (Table 4). Adjusted odds ratios for emergency room visits increased with the decrease in the pretransplant PROMIS-PF score [1.68 (95% confidence interval, 0.74–3.83) and 3.23 (1.34–7.79) in the mild and moderate/severe groups, respectively, with the normal category as the reference]. The risk of rehospitalization was significantly higher both in the mild and moderate/severe groups [adjusted odds ratios, 2.61 (1.16–5.90) and 2.53 (1.07–6.00)]. Multivariate regression analysis was not performed for other outcomes due to the small event numbers.

**TABLE 3:** Multivariate liner regression analysis of length of hospital stay and graft function.

PROMIS-PF score	Coefficient	95% CI	p-value
Length of stay (days, natural log-transformed)			
PROMIS-PF score (per 1-point increase)	−0.01	(−0.02, 0.00)	0.20
PROMIS-PF score category			
Normal (≥45): reference	ref		
Mild (40–<45)	0.14	(−0.05, 0.32)	0.15
Moderate/Severe (<40)	0.05	(−0.15, 0.25)	0.65
6-month eGFR (mL/min/1.73 m^2^)			
PROMIS-PF score (per 1-point increase)	0.0	(−0.5, 0.5)	0.90
PROMIS-PF score category			
Normal (≥45): reference	ref		
Mild (40–<45)	−5.1	(−12.7, 2.4)	0.18
Moderate/Severe (<40)	−0.2	(−8.3, 7.9)	0.96
12-month eGFR (mL/min/1.73 m^2^)			
PROMIS-PF score (per 1-point increase)	−0.1	(−0.6, 0.3)	0.61
PROMIS-PF score category			
Normal (≥45): reference	ref		
Mild (40–<45)	−2.3	(−9.7, 5.0)	0.53
Moderate/Severe (<40)	2.5	(−5.4, 10.4)	0.53

Linear regression was conducted for continuous PROMIS-PF scores and separately for the PROMIS-PF score category, adjusting for donor factors (age, donor type, donation after brain death/circulatory death, and cold ischemia time) and recipient variables (age, sex, race, Charleson Comorbidity Index, prior organ transplant, preemptive transplant, calculated panel reactive antibody, and lymphocyte-depleting antibody induction). Length of stay (days) was natural log-transformed to achieve a normal distribution.

CI, confidence interval; PROMIS PF, Patient-Reported Outcomes Measurement Information System Physical Function.

**TABLE 4:** Multivariate logistic regression analysis for emergency room visits and rehospitalization within 1-month posttransplant.

PROMIS-PF score	Odds ratio	95% CI	p-value
Emergency room visits within 1 month			
PROMIS-PF score (per 1-point increase)	0.94	(0.89, 0.99)	**0.023**
PROMIS-PF score category			
Normal (≥45): reference	ref		
Mild (40–<45)	1.68	(0.74, 3.83)	0.21
Moderate/Severe (<40)	3.23	(1.34, 7.79)	**0.009**
Rehospitalization within 1 month			
PROMIS-PF score (per 1-point increase)	0.94	(0.90, 1.00)	**0.033**
PROMIS-PF score category			
Normal (≥45): reference	ref		
Mild (40–<45)	2.61	(1.16, 5.90)	**0.021**
Moderate/Severe (<40)	2.53	(1.07, 6.00)	**0.035**

Associations between PROMIS-PF scores and events were analyzed using logistic regression, adjusting for the propensity scores that were calculated using donor factors (age, donor type, donation after brain death/circulatory death, and cold ischemia time) and recipient variables (age, sex, race, Charleson Comorbidity Index, prior organ transplant, preemptive transplant, calculated panel reactive antibody, and lymphocyte-depleting antibody induction). Logistic regression was performed for continuous PROMIS-PF scores and separately for the PROMIS-PF score category. Bold values denote statistically significant differences with p-values <0.05.

CI, confidence interval; PROMIS PF, Patient-Reported Outcomes Measurement Information System Physical Function.

### Comparison Between Recipients With and Without the PROMIS-PF Assessment

Given that only a subset of all transplant recipients completed the PROMIS-PF prior to their kidney transplantation, we compared the characteristics and transplant outcomes of recipients with and without the PROMIS-PF assessment (Supplementary Tables S5, S6). Compared to the recipients without the assessment, those with the assessment were more likely to have diabetes (31% vs. 38%) and exhibited higher Charlson Comorbidity Index values [median (IQR), 4 (2–5) vs. 5 (3–6)]. Recipients with the assessment more frequently received living-donor kidneys (34% vs. 44%) and less likely received the lymphocyte-depleting antibody induction (76% vs. 62%). Additionally, those with the score experienced higher rates of 1-month rehospitalization (22% vs. 38%) and 12-month mortality (3% vs. 6%). Other characteristics and outcomes did not show substantial differences between the groups.

## Discussion

Physical function is a significant and potentially modifiable prognostic factor among kidney transplant recipients [25, 26]. Low physical function is a major component of frailty, a condition common in kidney failure that is characterized by declines in physiological and cognitive states, associated with reduced physiologic reserve [1, 27]. Frailty is also associated with poor posttransplant outcomes and prehabilitation is being explored to improve outcomes [26, 28]. Therefore, it is imperative to establish simple and feasible physical function assessment tools to efficiently identify transplant candidates who may benefit from pretransplant prehabilitation. In this retrospective exploratory study, we investigated the associations between pretransplant PROMIS-PF scores and early transplant outcomes among kidney transplant recipients. While the pretransplant PROMIS-PF score was not associated with LOS or graft function, it was significantly associated with emergency room visits and rehospitalization within 1 month posttransplant. To our knowledge, this is the first study evaluating the PROMIS-PF CAT score in this patient population.

Previous studies have indicated that pretransplant low physical function and frailty are linked to longer LOS after kidney transplantation [29]. Lorenz et al. and Nastasi et al. conducted single-center studies that demonstrated a significant association between longer LOS and lower extremity functional impairment assessed using the Short Physical Performance Battery [30, 31]. In contrast, we found no association between the pretransplant PROMIS-PF score and LOS. This might be partly attributable to differences in the study periods because LOS has decreased over time [29]. Our study, covering kidney transplants between 2016 and 2023, reported a median LOS of 3 days. In comparison, the studies by Lorenz et al. and Nastasi et al., including kidney transplants before 2016, had median LOS of 4 and 8 days, respectively. Shorter LOS and improvements in patient care might have minimized LOS differences in our study. Additionally, variations in clinical practices and eligibility criteria for kidney transplantation between transplant centers could also explain the lack of association in the present study.

We did not find associations between the pretransplant PROMIS-PF score and graft function in this study. As serum creatinine concentration is influenced by muscle mass, the eGFR may be overestimated in recipients with lower physical function due to potentially reduced muscle mass. However, our findings are consistent with those of Lorenz et al., who also found no association between the Short Physical Performance Battery score and 12-month graft function measured via iothalamate clearance [30].

In line with previous studies that utilized different physical function measures such as the Kidney Disease Quality of Life Short Form and the Short Physical Performance Battery [30, 32, 33], the pretransplant PROMIS-PF score was significantly associated with posttransplant emergency room visits and rehospitalization. The odds ratios of emergency room visits increased with decreasing PROMIS-PF scores, indicating that the pretransplant PROMIS-PF score effectively captures these risks. The risk of rehospitalization was higher even in the mild group compared with the normal group. Our findings also align with those of Lorenz et al., who similarly reported a significant association between lower pretransplant PROMIS-PF 4-item short form scores with a higher risk of early rehospitalization after kidney transplantation [16]. Notably, they also found that the predictive value of the PROMIS-PF 4-item short form was comparable to frailty measures, including the physical frailty phenotype and the Short Physical Performance Battery. According to the study by Brodke et al., which documented the real-life physical ability indicated by the PROMIS-PF score, the physical function of score 45, distinguishing the normal from mild categories, corresponds to “Some difficulty with 2 h of physical labor and yard work; little difficulty with household chores and walking greater than 1 mile.” [34] Similarly, a score of 40, making the threshold of the mild and moderate/severe categories, corresponds to “Some difficulty with 2 h of physical labor, household chores, yard work, and walking greater than 1 mile.” These levels of pretransplant physical function may serve as a risk indicator for early posttransplant emergency room visits and rehospitalization.

Previous research on physical function and frailty demonstrated significant associations with delayed graft function, mortality, and graft survival [25, 28, 35]. Our study, however, could not evaluate these associations due to the small number of events observed. Larger-scale studies powered to detect clinician-driven outcomes are needed. However, these outcomes, such as graft function and survival, may not be as important to the patient as the quality-of-life health outcomes that are measured using PROMIS-PF. Measures such as PROMIS-PF allow patients to self-report their health status and subsequently one can assume they measure values and preferences that matter most to patients. A patient may care more about improving their ability to do physical labor, household chores, and yard work from much difficulty to little difficulty than whether they had delayed graft function. A preference elicitation study by Genie, et al. revealed that patient preferences among individuals with kidney failure are heterogeneous based on the patient’s age and duration of dialysis [36]. They found that graft survival did matter to patients and that patients were willing to wait an additional 29 months for transplantation for a graft that survived 5 more years (15 years vs. 10 years graft survival). Future preference elicitation studies should include quality of life outcomes and tradeoffs between clinical and graft survival outcomes among kidney transplant patients.

Given that PROMIS CAT demonstrates superior accuracy in measuring physical functioning across a broader range compared to other PROMs and achieves more precise results with fewer questions compared to most short forms, PROMIS CAT is considered particularly advantageous in the following situations: (1) assessing individuals with extremely poor health, (2) accurately measuring individuals with very good health, and (3) administering a small number of items [8, 11]. In situations with a broad range of anticipated physical functioning, CAT provides an accurate assessment with fewer items by tailoring questions to the individual’s functional level, avoiding asking irrelevant questions. This is relevant when assessing kidney transplant candidates. Furthermore, advantage (3) is a key feature for implementing universal and prospective physical function assessments in patients with kidney failure throughout the disease continuum, minimizing the burden on both patients and providers, particularly in high-volume centers. Our findings support the rationale for introducing PROMIS-PF CAT in such settings.

This study has several limitations. As this is a single-center retrospective study with a relatively small sample size and predominantly white patients, the generalizability of our findings may be limited. Selection bias is a potential concern given that PROMIS-PF tests were administered for clinical purposes and that only a portion of our patients were included in this study. Indeed, the comparison between recipients with and without the PROMIS-PF assessment suggested higher risk profiles among those with the assessment. Thus, the PROMIS-PF scores presented in this study may be worse than those of the general kidney transplant population. However, we believe that these relatively small differences do not have a substantial impact on our results. We were unable to adjust for all potential confounding factors due to the small sample size and limited event numbers. We also could not analyze important outcomes, such as mortality and graft failure. Additionally, because we had no standardized criteria for emergency room visits or admissions, these outcomes are subject to subjective decisions and may not be considered as strict research endpoints. While PROMIS CAT is suggested to provide more accurate results than fixed-length testing [11], we were unable to compare PROMIS-PF CAT with other physical function and frailty measures because we did not have these data.

In conclusion, a lower pretransplant PROMIS-PF CAT score was associated with a higher risk of emergency room visits and rehospitalization within 1 month posttransplant. Our findings indicate that PROMIS-PF could be a valuable physical function assessment tool in kidney transplant candidates. Further research with extended follow-up and larger sample sizes is needed to confirm the utility of the PROMIS-PF assessment in this population.

## Data Availability

The raw data supporting the conclusions of this article will be made available by the authors, without undue reservation.

## Abbreviations

CAT, computer adaptive testing; eGFR, estimated glomerular filtration rate; IQR, interquartile range; IRT, item response theory; LOS, length of transplant hospital stay; PROMs, Patient-reported outcome measures; PROMIS-PF, Patient-Reported Outcomes Measurement Information System Physical Function.
