# Supplementary material for: Associations of Pretransplant Patient-Reported Outcomes Measurement Information System Physical Function Score With Kidney Transplant Outcomes
Source: Transpl Int. 2025 Jan 29;38:13884. doi: 10.3389/ti.2025.13884 (PMC11813690; doi:10.3389/ti.2025.13884)
Supplement: Supplementary file 1 [file Table1.docx]

**Associations of Patient-Reported Outcomes Measurement Information System Physical Function Score with Kidney Transplant Outcomes**

**Supplementary Materials**

**Supplementary Tables**

Table S1. Reasons for emergency room visits and rehospitalization within 1 month posttransplant

Table S2. Multivariate liner regression analysis of length of hospital stay

Table S3. Multivariate liner regression analysis of 6-month eGFR (mL/min/1.73m2)

Table S4. Multivariate liner regression analysis of 12-month eGFR (mL/min/1.73m2)

Table S5. Patient characteristics according to the presence or absence of the PROMIS-PF measurement.

Table S6. Transplant outcomes according to the presence or absence of the PROMIS-PF measurement.

**Table S1. Reasons for emergency room visits and rehospitalization within 1 month posttransplant.**

| **Reasons for emergency room visits/rehospitalization** | **Total** | **PROMIS PF Category** | | |
| --- | --- | --- | --- | --- |
|  |  | **Normal (≥45)** | **Mild (40-<45)** | **Moderate/Severe (<40)** |
|  | N=154 | N=61 | N=52 | N=41 |
| **Emergency room visits** |  |  |  |  |
| Any emergency room visits within 1 month | 58 (38%) | 17 (28%) | 20 (38%) | 21 (51%) |
| Urinary tract infection | 9 (6%) | 4 (7%) | 0 (0%) | 5 (12%) |
| Other infections | 5 (3%) | 3 (5%) | 1 (2%) | 1 (2%) |
| Surgical wound problems | 11 (7%) | 3 (5%) | 5 (10%) | 3 (7%) |
| Other surgical complications (e.g., urinoma, lymphocele) | 2 (1%) | 0 (0%) | 1 (2%) | 1 (2%) |
| Graft dysfunction | 1 (1%) | 0 (0%) | 0 (0%) | 1 (2%) |
| Hyperkalemia | 3 (2%) | 0 (0%) | 2 (4%) | 1 (2%) |
| Cardiovascular | 4 (3%) | 1 (2%) | 1 (2%) | 2 (5%) |
| Gastrointestinal | 7 (5%) | 0 (0%) | 3 (6%) | 4 (10%) |
| Metabolic (glucose and electrolyte abnormalities except hyperkalemia) | 3 (2%) | 2 (3%) | 1 (2%) | 0 (0%) |
| Hypertension/hypotension | 5 (3%) | 1 (2%) | 4 (8%) | 0 (0%) |
| Others | 8 (5%) | 3 (5%) | 2 (4%) | 3 (7%) |
| **Rehospitalization** |  |  |  |  |
| Any rehospitalization within 1 month | 59 (38%) | 16 (26%) | 24 (46%) | 19 (46%) |
| Urinary tract infection | 8 (5%) | 3 (5%) | 1 (2%) | 4 (10%) |
| Other infections | 8 (5%) | 4 (7%) | 3 (6%) | 1 (2%) |
| Surgical wound problems | 6 (4%) | 1 (2%) | 4 (8%) | 1 (2%) |
| Other surgical complications (e.g., urinoma, lymphocele) | 4 (3%) | 0 (0%) | 1 (2%) | 3 (7%) |
| Graft dysfunction | 1 (1%) | 0 (0%) | 0 (0%) | 1 (2%) |
| Hyperkalemia | 3 (2%) | 0 (0%) | 2 (4%) | 1 (2%) |
| Cardiovascular | 3 (2%) | 1 (2%) | 1 (2%) | 1 (2%) |
| Gastrointestinal | 9 (6%) | 1 (2%) | 3 (6%) | 5 (12%) |
| Metabolic (glucose and electrolyte abnormalities except hyperkalemia) | 3 (2%) | 2 (3%) | 0 (0%) | 1 (2%) |
| Hypertension/hypotension | 5 (3%) | 0 (0%) | 5 (10%) | 0 (0%) |
| Others | 9 (6%) | 4 (7%) | 4 (8%) | 1 (2%) |

**Table S2. Multivariate liner regression analysis of length of hospital stay.**

| **Covariates** | **PROMIS-PF Continuous** | | | | **PROMIS-PF Category** | | | |
| --- | --- | --- | --- | --- | --- | --- | --- | --- |
|  | **Coef.** | **95% CI** | **p-value** | **Stan coef.** | **Coef.** | **95% CI** | **p-value** | **Stan coef.** |
| **Recipient** |  |  |  |  |  |  |  |  |
| PROMIS-PF score (per 1-point increase) | -0.01 | (-0.02, 0.00) | 0.20 | -0.107 |  |  |  |  |
| PROMIS-PF score category |  |  |  |  |  |  |  |  |
| Normal (≥45): reference |  |  |  |  | ref |  |  |  |
| Mild (40-<45) |  |  |  |  | 0.14 | (-0.05, 0.32) | 0.15 | 0.130 |
| Moderate/Severe (<40) |  |  |  |  | 0.05 | (-0.15, 0.25) | 0.65 | 0.041 |
| Age (per 1-year increase) | 0.00 | (-0.00, 0.01) | 0.26 | 0.105 | 0.00 | (-0.00, 0.01) | 0.25 | 0.108 |
| Sex (male vs female) | 0.17 | (-0.00, 0.35) | 0.054 | 0.172 | 0.17 | (-0.01, 0.35) | 0.058 | 0.169 |
| Race White (yes vs no) | -0.09 | (-0.28, 0.10) | 0.34 | -0.086 | -0.09 | (-0.28, 0.10) | 0.36 | -0.085 |
| Prior organ transplantation (yes vs no) | -0.19 | (-0.51, 0.13) | 0.24 | -0.114 | -0.21 | (-0.53, 0.11) | 0.19 | -0.126 |
| Preemptive transplant (yes vs no) | 0.05 | (-0.15, 0.25) | 0.62 | 0.044 | 0.03 | (-0.17, 0.23) | 0.78 | 0.025 |
| Calculated panel reactive antibody (per 1% increase) | 0.00 | (-0.00, 0.01) | 0.082 | 0.183 | 0.00 | (-0.00, 0.01) | 0.082 | 0.184 |
| Charlson Comorbidity Index category (tertile) |  |  |  |  |  |  |  |  |
| 2-4: reference | ref |  |  |  | ref |  |  |  |
| 5-6 | 0.03 | (-0.17, 0.22) | 0.80 | 0.024 | 0.02 | (-0.17, 0.22) | 0.82 | 0.021 |
| 7-16 | 0.12 | (-0.10, 0.34) | 0.27 | 0.105 | 0.12 | (-0.09, 0.34) | 0.26 | 0.108 |
| Lymphocyte depleting induction (yes vs no) | 0.10 | (-0.08, 0.29) | 0.28 | 0.100 | 0.12 | (-0.07, 0.30) | 0.22 | 0.116 |
| **Donor** |  |  |  |  |  |  |  |  |
| Donor type |  |  |  |  |  |  |  |  |
| Living: reference | ref |  |  |  | ref |  |  |  |
| Deceased, brain death | 0.19 | (-0.06, 0.45) | 0.14 | 0.191 | 0.22 | (-0.04, 0.47) | 0.10 | 0.212 |
| Deceased, circulatory death | 0.27 | (-0.03, 0.58) | 0.074 | 0.221 | 0.30 | (-0.00, 0.60) | 0.053 | 0.242 |
| Age (per 1-year increase) | 0.00 | (-0.01, 0.00) | 0.81 | -0.021 | 0.00 | (-0.01, 0.01) | 0.94 | -0.006 |
| Cold ischemia time (per 1-hour increase) | -0.01 | (-0.02, 0.01) | 0.35 | -0.118 | -0.01 | (-0.02, 0.01) | 0.23 | -0.153 |

Length of stay (days) was natural log-transformed to achieve a normal distribution.

CI, confidence interval; Coef., coefficient; PROMIS PF, Patient-Reported Outcomes Measurement Information System Physical Function; Stan coef., standardized coefficient.

**Table S3. Multivariate liner regression analysis of 6-month eGFR (mL/min/1.73m2).**

| **Covariates** | **PROMIS-PF Continuous** | | | | **PROMIS-PF Category** | | | |
| --- | --- | --- | --- | --- | --- | --- | --- | --- |
|  | **Coef.** | **95% CI** | **p-value** | **Stan coef.** | **Coef.** | **95% CI** | **p-value** | **Stan coef.** |
| **Recipient** |  |  |  |  |  |  |  |  |
| PROMIS-PF score (per 1-point increase) | 0.0 | (-0.5, 0.5) | 0.90 | 0.010 |  |  |  |  |
| PROMIS-PF score category |  |  |  |  |  |  |  |  |
| Normal (≥45): reference |  |  |  |  | ref |  |  |  |
| Mild (40-<45) |  |  |  |  | -5.1 | (-12.7, 2.4) | 0.18 | -0.116 |
| Moderate/Severe (<40) |  |  |  |  | -0.2 | (-8.3, 7.9) | 0.96 | -0.004 |
| Age (per 1-year increase) | -0.3 | (-0.6, -0.1) | **0.008** | -0.241 | -0.4 | (-0.6, -0.1) | **0.006** | -0.248 |
| Sex (male vs female) | -1.9 | (-9.1, 5.3) | 0.60 | -0.045 | -2.0 | (-9.1, 5.2) | 0.59 | -0.046 |
| Race White (yes vs no) | -1.1 | (-8.9, 6.6) | 0.77 | -0.025 | -1.2 | (-8.9, 6.6) | 0.77 | -0.026 |
| Prior organ transplantation (yes vs no) | -2.3 | (-15.3, 10.7) | 0.73 | -0.032 | -2.2 | (-15.1, 10.7) | 0.73 | -0.032 |
| Preemptive transplant (yes vs no) | 1.9 | (-6.3, 10.2) | 0.64 | 0.040 | 2.3 | (-5.9, 10.5) | 0.58 | 0.047 |
| Calculated panel reactive antibody (per 1% increase) | -0.1 | (-0.2, 0.0) | 0.12 | -0.157 | -0.1 | (-0.2, 0.0) | 0.10 | -0.167 |
| Charlson Comorbidity Index category (tertile) |  |  |  |  |  |  |  |  |
| 2-4: reference | ref |  |  |  | ref |  |  |  |
| 5-6 | -0.9 | (-8.9, 7.1) | 0.83 | -0.019 | -0.6 | (-8.6, 7.4) | 0.88 | -0.013 |
| 7-16 | 2.3 | (-6.5, 11.1) | 0.61 | 0.048 | 2.7 | (-6.0, 11.5) | 0.54 | 0.056 |
| Lymphocyte depleting induction (yes vs no) | 0.0 | (-7.7, 7.6) | 0.99 | -0.001 | -0.6 | (-8.2, 7.1) | 0.88 | -0.013 |
| **Donor** |  |  |  |  |  |  |  |  |
| Donor type |  |  |  |  |  |  |  |  |
| Living: reference | ref |  |  |  | ref |  |  |  |
| Deceased, brain death | 0.0 | (-10.5, 10.6) | 1.00 | 0.000 | -0.5 | (-10.9, 10.0) | 0.93 | -0.011 |
| Deceased, circulatory death | -2.2 | (-14.6, 10.1) | 0.72 | -0.042 | -2.9 | (-15.3, 9.4) | 0.64 | -0.055 |
| Age (per 1-year increase) | -0.4 | (-0.6, -0.2) | **0.001** | -0.284 | -0.4 | (-0.6, -0.2) | **0.001** | -0.289 |
| Cold ischemia time (per 1-hour increase) | 0.1 | (-0.5, 0.7) | 0.78 | 0.033 | 0.2 | (-0.5, 0.8) | 0.63 | 0.059 |

CI, confidence interval; Coef., coefficient; PROMIS PF, Patient-Reported Outcomes Measurement Information System Physical Function; Stan coef., standardized coefficient.

**Table S4. Multivariate liner regression analysis of 12-month eGFR (mL/min/1.73m2).**

| **Covariates** | **PROMIS-PF Continuous** | | | | **PROMIS-PF Category** | | | |
| --- | --- | --- | --- | --- | --- | --- | --- | --- |
|  | **Coef.** | **95% CI** | **p-value** | **Stan coef.** | **Coef.** | **95% CI** | **p-value** | **Stan coef.** |
| **Recipient** |  |  |  |  |  |  |  |  |
| PROMIS-PF score (per 1-point increase) | -0.1 | (-0.6, 0.3) | 0.61 | -0.042 |  |  |  |  |
| PROMIS-PF score category |  |  |  |  |  |  |  |  |
| Normal (≥45): reference |  |  |  |  | ref |  |  |  |
| Mild (40-<45) |  |  |  |  | -2.3 | (-9.7, 5.0) | 0.53 | -0.055 |
| Moderate/Severe (<40) |  |  |  |  | 2.5 | (-5.4, 10.4) | 0.53 | 0.055 |
| Age (per 1-year increase) | -0.3 | (-0.6, -0.1) | **0.012** | -0.227 | -0.3 | (-0.6, -0.1) | **0.012** | -0.230 |
| Sex (male vs female) | -0.1 | (-7.1, 6.9) | 0.98 | -0.003 | -0.1 | (-7.1, 6.8) | 0.97 | -0.003 |
| Race White (yes vs no) | 0.8 | (-6.7, 8.4) | 0.83 | 0.019 | 0.8 | (-6.7, 8.4) | 0.83 | 0.019 |
| Prior organ transplantation (yes vs no) | -4.0 | (-16.6, 8.6) | 0.53 | -0.059 | -4.1 | (-16.6, 8.5) | 0.52 | -0.060 |
| Preemptive transplant (yes vs no) | 4.5 | (-3.5, 12.5) | 0.27 | 0.095 | 4.5 | (-3.5, 12.4) | 0.27 | 0.095 |
| Calculated panel reactive antibody (per 1% increase) | -0.1 | (-0.2, 0.0) | 0.17 | -0.138 | -0.1 | (-0.2, 0.0) | 0.15 | -0.147 |
| Charlson Comorbidity Index category (tertile) |  |  |  |  |  |  |  |  |
| 2-4: reference | ref |  |  |  | ref |  |  |  |
| 5-6 | 2.4 | (-5.3, 10.2) | 0.54 | 0.055 | 2.5 | (-5.2, 10.3) | 0.52 | 0.058 |
| 7-16 | 2.6 | (-5.9, 11.2) | 0.54 | 0.056 | 3.0 | (-5.6, 11.5) | 0.49 | 0.063 |
| Lymphocyte depleting induction (yes vs no) | 2.4 | (-5.0, 9.9) | 0.51 | 0.059 | 2.2 | (-5.3, 9.6) | 0.57 | 0.052 |
| **Donor** |  |  |  |  |  |  |  |  |
| Donor type |  |  |  |  |  |  |  |  |
| Living: reference | ref |  |  |  | ref |  |  |  |
| Deceased, brain death | 1.8 | (-8.4, 12.0) | 0.73 | 0.043 | 1.5 | (-8.7, 11.8) | 0.77 | 0.037 |
| Deceased, circulatory death | 0.3 | (-11.7, 12.3) | 0.97 | 0.005 | -0.3 | (-12.4, 11.7) | 0.96 | -0.007 |
| Age (per 1-year increase) | -0.4 | (-0.6, -0.2) | **<0.001** | -0.298 | -0.4 | (-0.6, -0.2) | **<0.001** | -0.299 |
| Cold ischemia time (per 1-hour increase) | -0.1 | (-0.7, 0.5) | 0.71 | -0.045 | -0.1 | (-0.7, 0.5) | 0.82 | -0.028 |

CI, confidence interval; Coef., coefficient; PROMIS PF, Patient-Reported Outcomes Measurement Information System Physical Function; Stan coef., standardized coefficient.

**Table S5. Patient characteristics according to the presence or absence of the PROMIS-PF measurement.**

| **Characteristic** | **Total** | **PROMIS-PF (-)** | **PROMIS-PF (+)** | **p-value** |
| --- | --- | --- | --- | --- |
|  | N=1,012 | N=858 | N=154 |  |
| **Recipient** |  |  |  |  |
| Age (years) | 50 (15) | 50 (15) | 52 (14) | 0.17 |
| Sex |  |  |  | 0.58 |
| Female | 394 (39%) | 331 (39%) | 63 (41%) |  |
| Male | 618 (61%) | 527 (61%) | 91 (59%) |  |
| Body mass index (kg/m2) | 28.0 (5.2) | 27.9 (5.2) | 28.2 (5.2) | 0.60 |
| Race |  |  |  | **0.009** |
| White | 688 (68%) | 583 (68%) | 105 (68%) |  |
| Black | 20 (2%) | 15 (2%) | 5 (3%) |  |
| Hispanic | 195 (19%) | 174 (20%) | 21 (14%) |  |
| Asian | 35 (3%) | 30 (3%) | 5 (3%) |  |
| American Indian/Alaska Native | 33 (3%) | 29 (3%) | 4 (3%) |  |
| Native Hawaiian/other Pacific Islander | 36 (4%) | 23 (3%) | 13 (8%) |  |
| Multiracial | 5 (0%) | 4 (0%) | 1 (1%) |  |
| History of diabetes | 325 (32%) | 266 (31%) | 59 (38%) | 0.074 |
| Prior organ transplantation | 88 (9%) | 73 (9%) | 15 (10%) | 0.62 |
| Dialysis duration |  |  |  | **0.042** |
| Preemptive | 198 (20%) | 160 (19%) | 38 (25%) |  |
| ≤1 year | 153 (15%) | 128 (15%) | 25 (16%) |  |
| 1-3 years | 253 (25%) | 221 (26%) | 32 (21%) |  |
| 3-5 years | 246 (24%) | 219 (26%) | 27 (18%) |  |
| >5 years | 162 (16%) | 130 (15%) | 32 (21%) |  |
| Cause of kidney failure |  |  |  | **0.001** |
| Diabetes | 278 (27%) | 228 (27%) | 50 (32%) |  |
| Hypertension | 147 (15%) | 128 (15%) | 19 (12%) |  |
| Glomerulonephritis | 210 (21%) | 188 (22%) | 22 (14%) |  |
| Cystic disease | 100 (10%) | 73 (9%) | 27 (18%) |  |
| Others | 277 (27%) | 241 (28%) | 36 (23%) |  |
| Hepatitis B virus core antibody | 73 (8%) | 61 (8%) | 12 (9%) | 0.79 |
| Hepatitis C virus antibody | 15 (1%) | 10 (1%) | 5 (3%) | 0.051 |
| Human immunodeficiency virus antibody | 2 (0%) | 0 (0%) | 2 (1%) | **<0.001** |
| Charlson Comorbidity Index at transplant | 4 (2-5) | 4 (2-5) | 5 (3-6) | **<0.001** |
| Calculated panel reactive antibody (%) | 0 (0-0) | 0 (0-0) | 0 (0-0) | 0.50 |
| Human leucocyte antigen mismatch |  |  |  | 0.22 |
| 0 | 50 (5%) | 42 (5%) | 8 (5%) |  |
| 1 | 27 (3%) | 20 (2%) | 7 (5%) |  |
| 2 | 71 (7%) | 55 (6%) | 16 (10%) |  |
| 3 | 186 (18%) | 163 (19%) | 23 (15%) |  |
| 4 | 249 (25%) | 218 (25%) | 31 (20%) |  |
| 5 | 301 (30%) | 252 (29%) | 49 (32%) |  |
| 6 | 128 (13%) | 108 (13%) | 20 (13%) |  |
| Induction immunosuppression | |  |  |  |
| Lymphocyte depleting induction | 749 (74%) | 654 (76%) | 95 (62%) | **<0.001** |
| Anti-thymocyte globulin | 537 (53%) | 481 (56%) | 56 (36%) | **<0.001** |
| Alemtuzumab | 224 (22%) | 183 (21%) | 41 (27%) | 0.15 |
| Basiliximab | 45 (4%) | 35 (4%) | 10 (6%) | 0.18 |
| Maintenaice immunosuppression at discharge | |  |  |  |
| Tacrolimus | 908 (90%) | 768 (90%) | 140 (91%) | 0.60 |
| Cyclosporine | 19 (2%) | 17 (2%) | 2 (1%) | 0.57 |
| Everolimus | 5 (0%) | 4 (0%) | 1 (1%) | 0.77 |
| Belatacept | 167 (17%) | 148 (17%) | 19 (12%) | 0.13 |
| Mycophenolate | 1,012 (100%) | 858 (100%) | 154 (100%) | - |
| Steroids | 1,009 (100%) | 855 (100%) | 154 (100%) | 0.46 |
| **Donor** |  |  |  |  |
| Donor type |  |  |  | **0.028** |
| Living donor | 361 (36%) | 294 (34%) | 67 (44%) |  |
| Deceased donor | 651 (64%) | 564 (66%) | 87 (56%) |  |
| Age (years) | 37 (16) | 36 (17) | 39 (15) | 0.053 |
| Sex |  |  |  | 0.56 |
| Female | 484 (48%) | 407 (47%) | 77 (50%) |  |
| Male | 528 (52%) | 451 (53%) | 77 (50%) |  |
| Terminal serum creatinine (mg/dL) | 0.93 (0.51) | 0.93 (0.52) | 0.96 (0.48) | 0.53 |
| Kidney Donor Profile Index | 38 (25) | 38 (25) | 39 (23) | 0.60 |
| Donation after circulatory death | 271 (42%) | 241 (43%) | 30 (34%) | 0.15 |
| Donor kidney on-pump | 639 (63%) | 553 (64%) | 86 (56%) | **0.041** |
| Cold ischemia time (hours) | 10 (8) | 11 (8) | 9 (8) | **0.018** |

Values are expressed as mean (standard deviation), median (interquartile range), or number (%).

Continuous variables were compared via the t-tests or the Mann-Whitney U tests. Chi-square tests were used for categorical variables.

**Table S6. Transplant outcomes according to the presence or absence of the PROMIS-PF measurement.**

| **Outcome** | **Total** | **PROMIS-PF (-)** | **PROMIS-PF (+)** | **p-value** |
| --- | --- | --- | --- | --- |
|  | N=1,012 | N=858 | N=154 |  |
| Length of hospital stay (days) | 3 (3-4) | 3 (3-4) | 3 (3-4) | 0.23 |
| Length of hospital stay ≥7 days | 120 (12%) | 107 (12%) | 13 (8%) | 0.15 |
| Delayed graft function | 22 (2%) | 16 (2%) | 6 (4%) | 0.11 |
| Any emergency room visit within 1 month | 354 (35%) | 296 (34%) | 58 (38%) | 0.45 |
| Any rehospitalization within 1 month | 248 (25%) | 189 (22%) | 59 (38%) | **<0.001** |
| 6-month eGFR (mL/min/1.73m2) | 66 (22) | 66 (22) | 63 (21) | 0.10 |
| 12-month eGFR (mL/min/1.73m2) | 66 (22) | 66 (22) | 63 (20) | 0.11 |
| 6-month death-censored graft failure | 13 (1%) | 12 (1%) | 1 (1%) | 0.45 |
| 12-month death-censored graft failure | 18 (2%) | 16 (2%) | 2 (1%) | 0.62 |
| 6-month mortality | 23 (2%) | 21 (2%) | 2 (1%) | 0.38 |
| 12-month mortality | 34 (3%) | 24 (3%) | 10 (6%) | **0.019** |

Values are expressed as mean (standard deviation), median (interquartile range), or number (%).

Continuous variables were compared via the t-tests or the Mann-Whitney U tests. Chi-square tests were used for categorical variables.
